# Supplementary material for: The non-coding RNA BC1 regulates experience-dependent structural plasticity and learning
Source: Nat Commun. 2017 Aug 17;8:293. doi: 10.1038/s41467-017-00311-2 (PMC5561022; doi:10.1038/s41467-017-00311-2)
Supplement: Supplementary file 1 — Supplementary Information [file 41467_2017_311_MOESM1_ESM.pdf]

File Name: Supplementary Information
Description: Supplementary Figures, Supplementary Table and Supplementary References

File Name: Supplementary Movie 1
Description: Serial block face scanning electron microscopy (SBF-SEM) imaging followed by 3D reconstruction of barrel cortex synapses in WT mice. The serial images were aligned using DigitalMicrograph software and reconstructed using Imaris software. Synapse ultrastructures are highlighted with different colors: dendritic spines (blue), presynaptic terminal (green), synaptic vesicles (magenta), postsynaptic density (yellow), active zone (orange).

File Name: Supplementary Movie 2
Description: Serial block face scanning electron microscopy (SBF-SEM) imaging followed by 3D reconstruction of barrel cortex synapses in *BC1* KO mice. The serial images were aligned using DigitalMicrograph software and reconstructed using Imaris software. Synapse ultrastructures are highlighted with different colors: dendritic spines (blue), presynaptic terminal (green), synaptic vesicles (magenta), postsynaptic density (yellow), active zone (orange).

File Name: Peer Review File
Description:

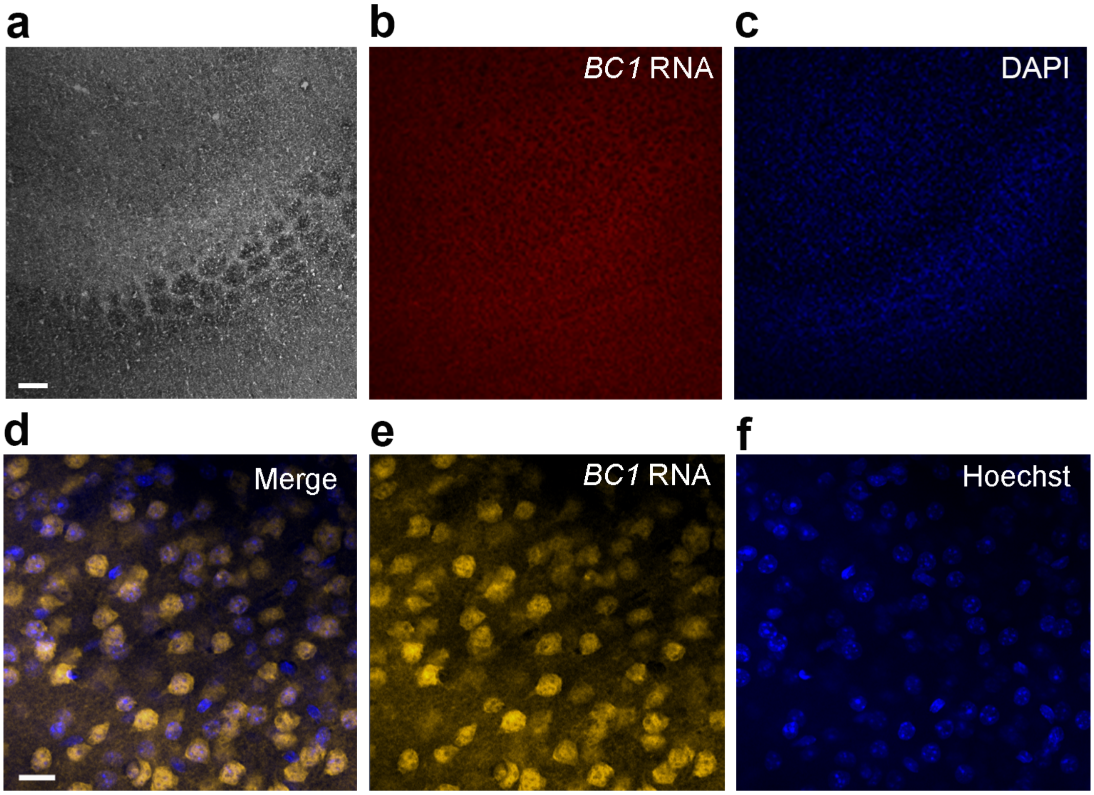

Supplementary Figure 1. *BC1* RNA is expressed in young adult mouse somatosensory cortex. (**a**) Bright field image of the mouse somatosensory cortex upon cytochrome oxidase (CO) activity. Anatomical structures in layer 4 termed ‘‘barrels’’ are shown. Scale bar = 200 μm (**b**) FISH detecting *BC1* RNA in the barrels using antisense RNA probe. (**c**) Cell nuclei stained with DAPI. (**d-f**) FISH detecting *BC1* RNA in a coronal section of the barrel cortex (enlargement 63x). *BC1* RNA signal (yellow), nuclear signal (Hoechst staining blue). Scale bar = 20 μm.

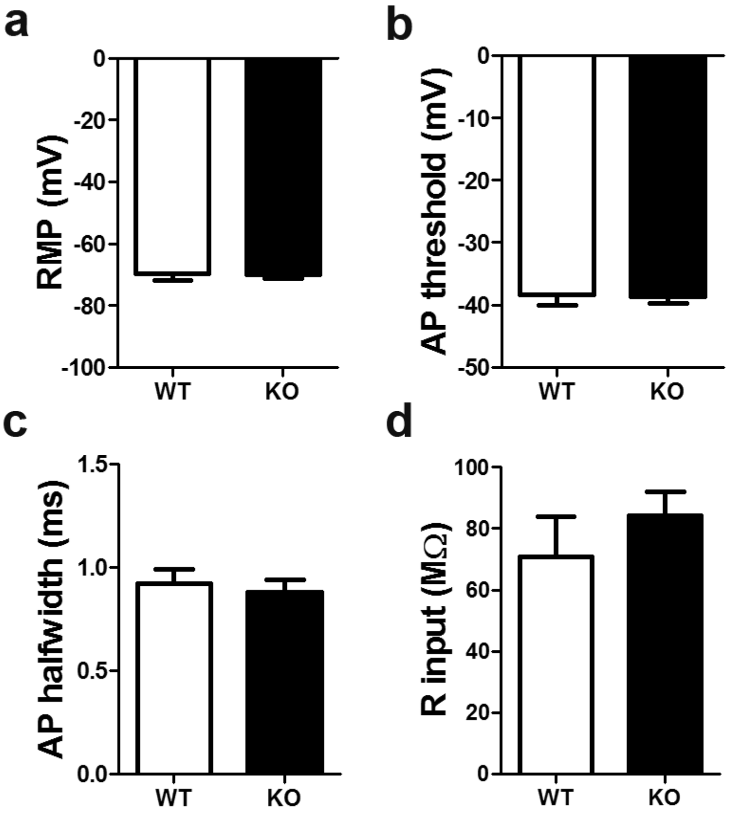

Supplementary Figure 2. *BC1* KO pyramidal neurons show normal basic action potential firing and membrane properties. (**a-d**) Resting membrane potential (RMP), action potential (AP) threshold, AP halfwidth, and input resistance (R) in layer 2/3 pyramidal neurons from the barrel cortex were not affected by the absence of *BC1* RNA (mean ± s.e.m., n = 13 WT and 17 KO neurons).

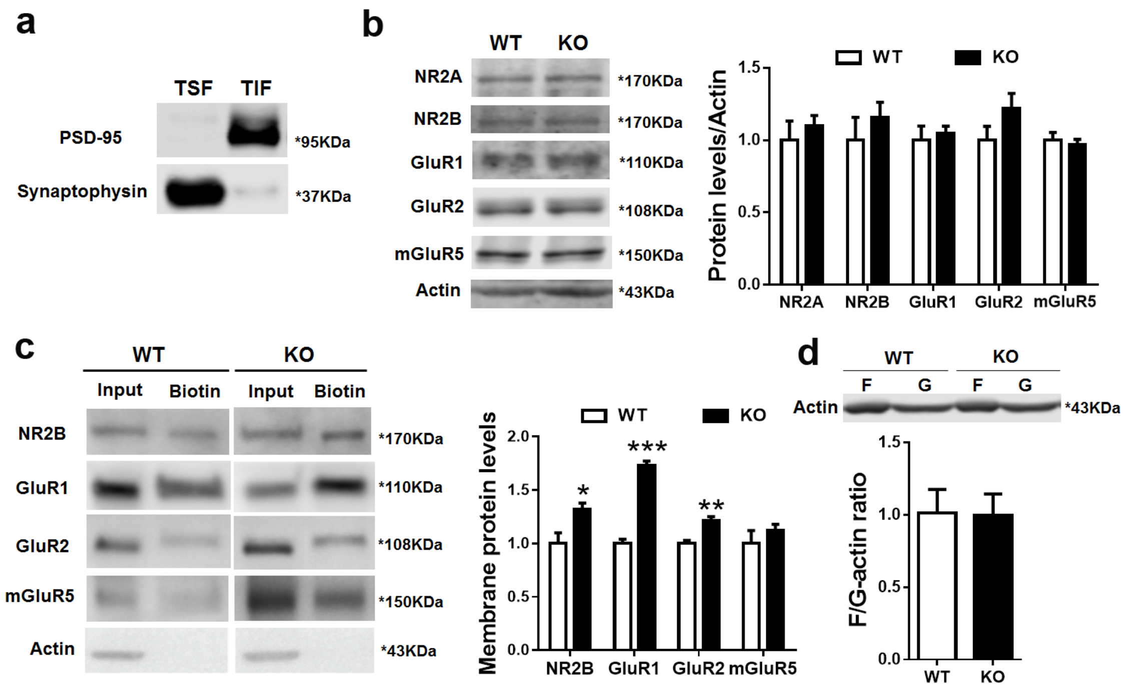

Supplementary Figure 3. *BC1* KO neurons have increased membrane levels of glutamate receptors. (**a**) Levels of PSD-95 and Synaptophysin in Triton-soluble fraction (TSF) and Triton-insoluble fraction (TIF) from a cortical lysate. (**b**) Left, representative immunoblots of protein levels in whole cortical lysates from WT and *BC1* KO neurons. Right, quantification of protein levels. Values are expressed as ratio (fold of WT) of NR2A, NR2B, GluR1, GluR2, and mGluR5 levels over Actin (mean ± s.e.m., n = 6 WT and 4 KO mice). (**c**) Left, representative immunoblots of total (input) and biotinylated proteins from WT and *BC1* KO primary cortical neurons. Right, quantification of protein levels. Values are expressed as fold of WT (**P* = 0.0253, ***P* = 0.0015, ****P* < 0.0001, two-tailed t-test, mean ± s.e.m., n = 5 mice). Actin was only detected in the input because the biotinylation assay allows to visualize membrane proteins (**d**) Top, representative Western blotting of filamentous (F) and globular (G) actin from WT and *BC1* KO cortical tissue lysates. Bottom, quantification of F/G-actin ratio (mean ± s.e.m., n = 6 WT and 5 KO mice).

```
BC1 RNA     59 UGGGUCCCGGAACGCGAACGAUCCG 35
               ||||..||||||| |||. ||||||                       20/25 (80.0%)
Arc       2596 ACCCCAGGCCUUG-GCUC-CUAGGC 2618


BC1 RNA     27 GAUGGUGACUCGAUUUAGGG 8
               ||||...|||||||.|||||                            16/20 (80.0%)
αCaMKII   3532 CUACAUGUGAGCUAUAUCCC 3551


BC1 RNA     28 AGAUGGUGAC-UCGAUUU 12
               ||||||.||| |||||||                              16/18 (88.9%)
MAP1B     5510 UCUACCUCUGCAGCUAAA 5527


BC1 RNA     18 UCGAUUUAGGGGUUGGGG 1
               ||||.|..|||||.||||                              14/18 (77.8%)
PSD-95       3 AGCUCAUGCCCCAGCCCC 20


BC1 RNA     44 GAACGAUCCGUUC-GCGAGAUGGUGACUCG 16
               |.||||..||||| ||||||||| ||||.|                  24/30 (80.0%)
NR2B      4870 CAUGCUCAGCAAGUCGCUCUACC-CUGACC 4898


BC1 RNA     67 UCCUGGCUUGGGUCCCGGAACGCGAACG 40
               ||||.|.|||||||  |.||||||||.|                    22/28 (78.6%)
mGluR5     138 AGGAGCCAACCCAG--CGUUGCGCUUCC 163


BC1 RNA     77 AGGUCUCGACUCCUGGCUUGGGUCCCGGAACGCGAAC 41
               |||.||..|.|||||. |..||||.||..||.||.||           24/37 (64.9%)
GluR1     2790 UCCGGAAGUAAGGACA-AGACCAGUGCUCUGAGCCUG 2825
```

Supplementary Figure 4. *BC1* RNA shows sequence complementarity with several dendritic mRNAs. Mouse *BC1* RNA shows short sequence complementarity with regions of *Arc*, *αCaMKII*, *MAP1B*, *NR2B*, *mGluR5* and *PSD-95* mRNAs. Sequences were analyzed using the EMBOSS matcher online tool (http://www.ebi.ac.uk/). The parameter settings were: "gap open", 16; "gap extend", 8; and "alternatives matches", 3. The match with the highest homology (reported on the right) is shown. Use of the matcher algorithm highlights new regions in *Arc*, *αCaMKII* and *MAP1B* mRNAs in addition to those reported and verified[1]; annealing of these new mRNA regions to *BC1* RNA has not yet been verified experimentally.

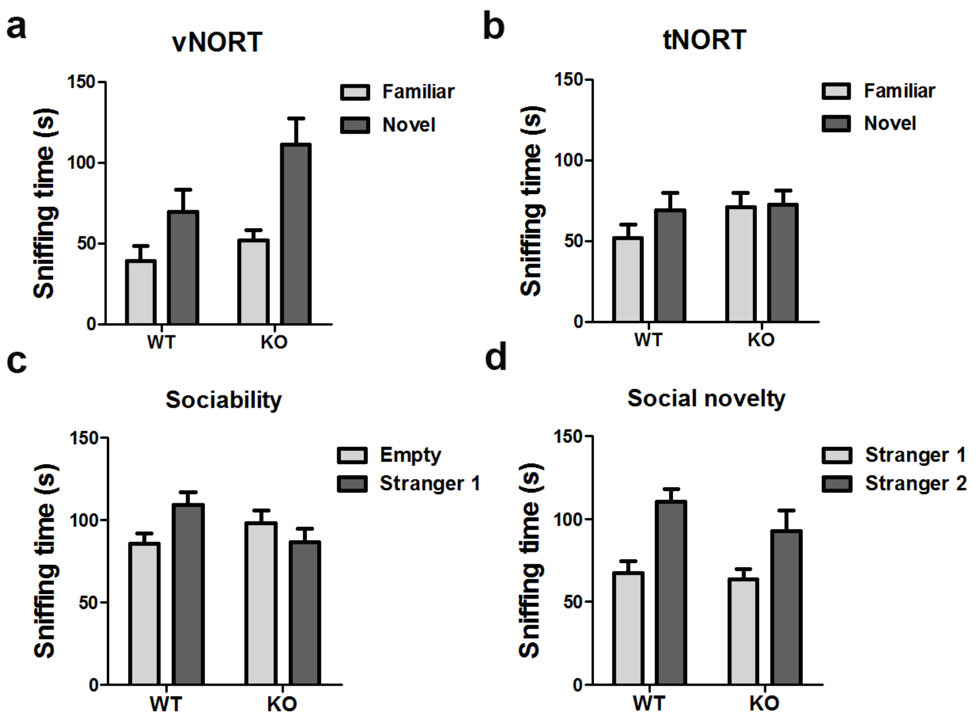

Supplementary Figure 5. *BC1* KO mice show deficits in the texture NORT (tNORT) and sociability. (**a-b**) Total sniffing time with novel and familiar objects in the visual novel object recognition test (vNORT) and tNORT (P = 0.007 for object effect and P = 0.0293 for genotype effect in vNORT, P > 0.05 for both factors in tNORT two-way ANOVA, mean ± s.e.m., n = 11 WT and 10 KO mice). The increased tendency on object preference in WT mice reaches statistical significance after normalization for the total exploration time (preference index, Fig. 7a main text). (**c**) Sociability. Total sniffing time in the three-chamber test (P = 0.0241 genotype x stranger interaction in sociability, two-way ANOVA, mean ± s.e.m., n = 17 WT and 11 KO mice). *BC1* KO showed no preference for stranger 1 (P > 0.05 in a post-hoc Student's t-test). (**d**) Social novelty. Total sniffing time in the three-chamber test. Both genotype equally preferred to explore the novel stranger 2 (P < 0.001 for stranger effect in social novelty, and P > 0.05 for genotype effect, two-way ANOVA, mean ± s.e.m., n = 17 WT and 11 KO mice).

Supplementary Figure 6. Full gel scans of Western blots shown in the main article.
Each blot is labeled with the figure-panel number reported in the main figure.
Please note that the apparently different molecular weight of some protein across experiments might be due to differences in SDS-PAGE running conditions.

Figure 4a

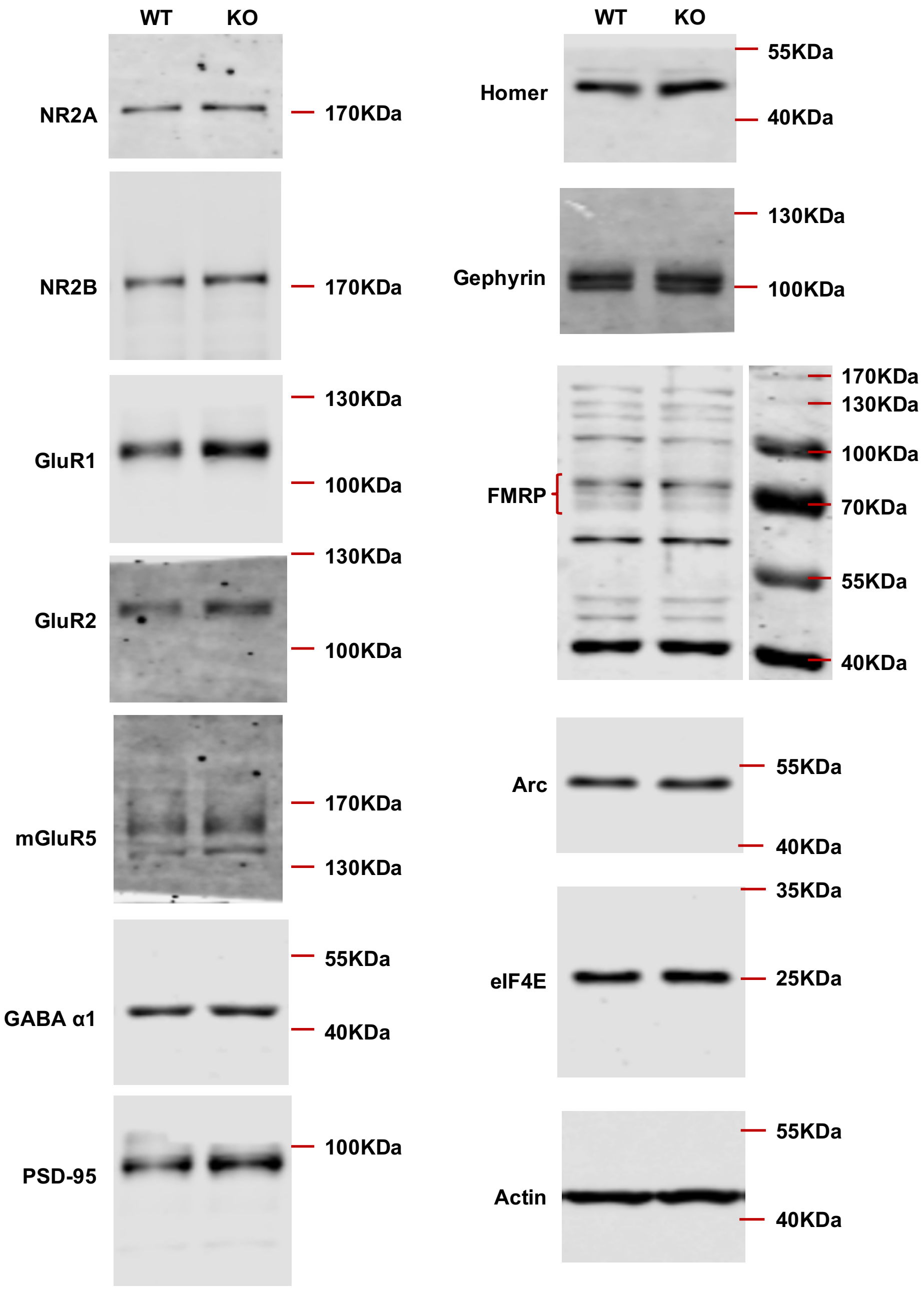

Figure 4b

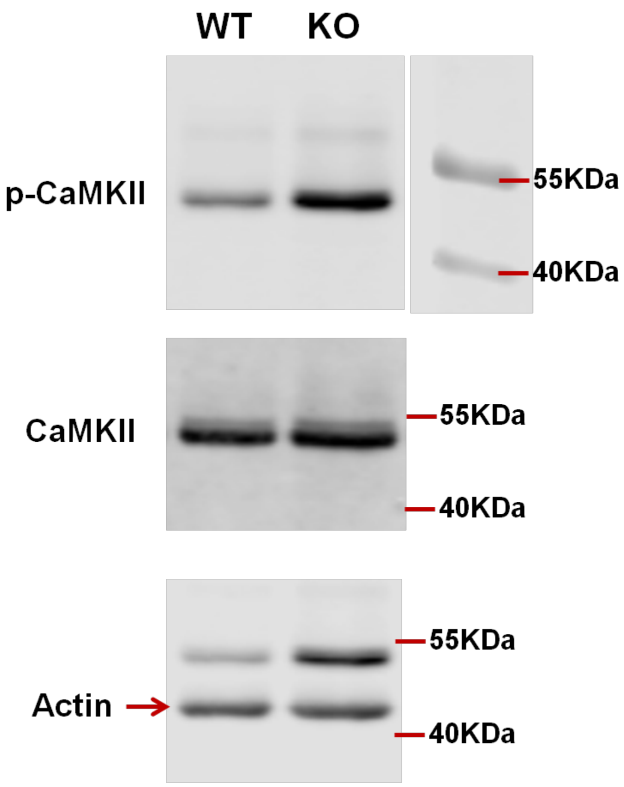

WT
KO
p-CaMKII
55KDa
40KDa
CaMKII
55KDa
40KDa
Actin
55KDa
40KDa

Figure 4c

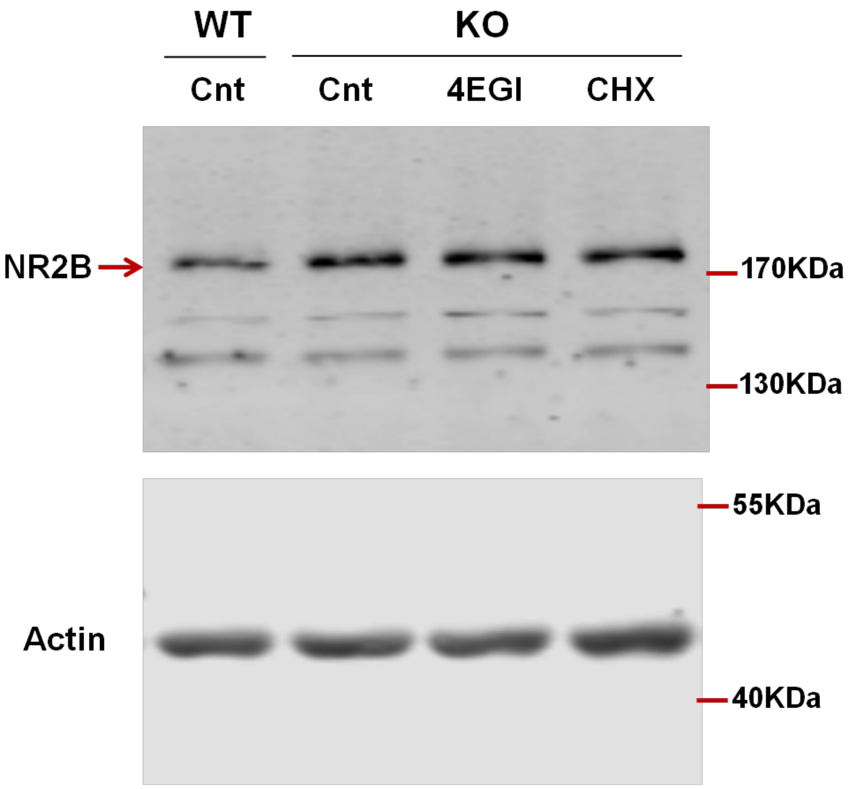

Figure 4d

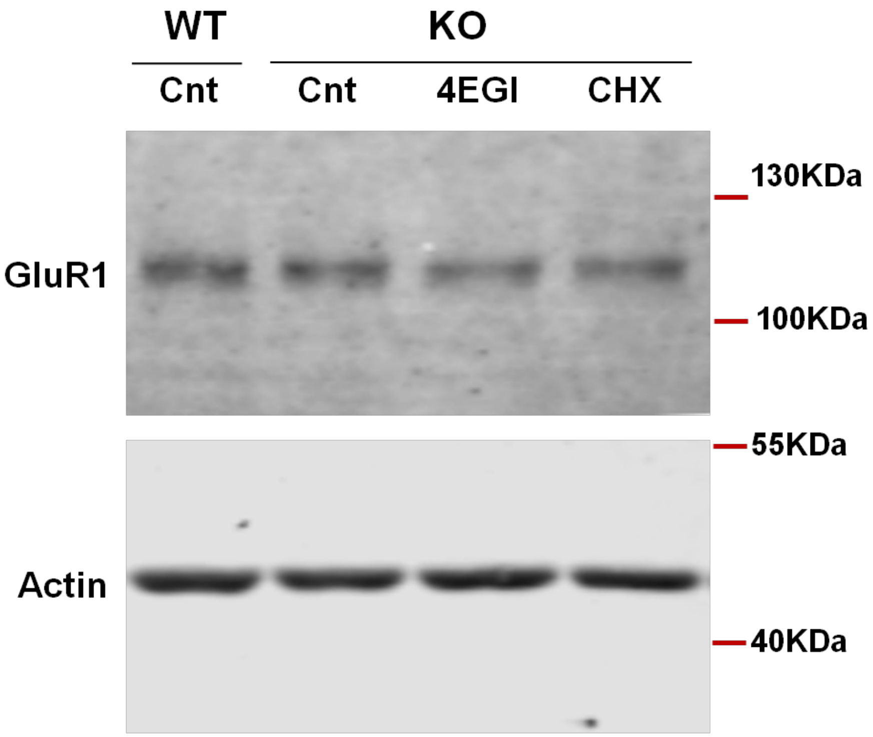

Figure 4e

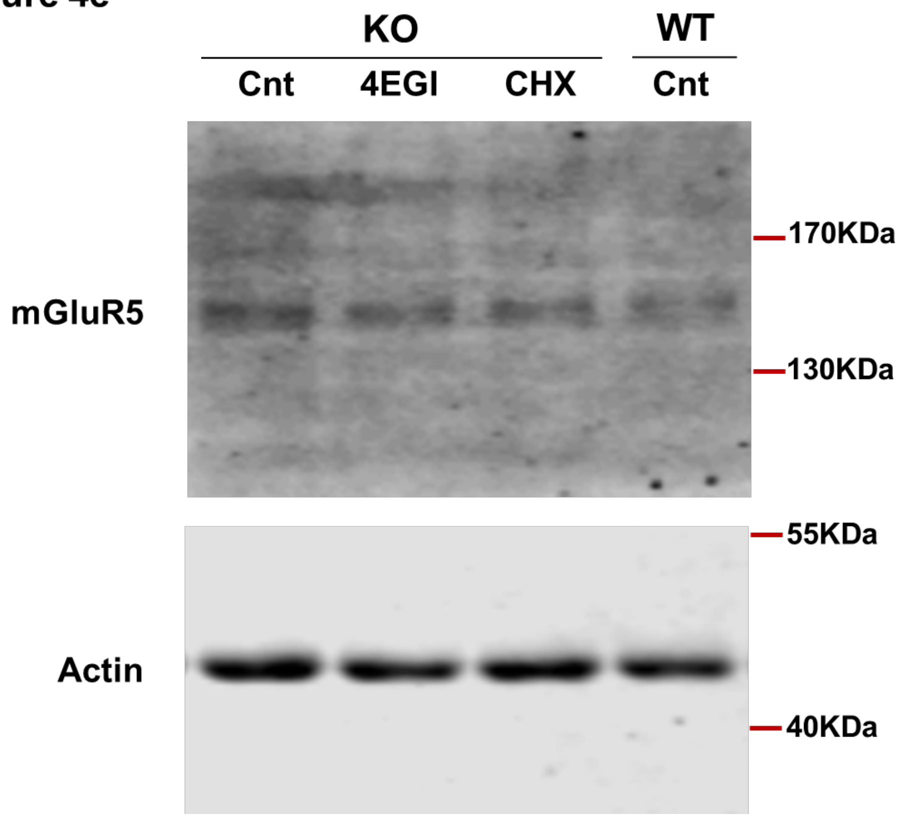
KO
WT
Cnt
4EGI
CHX
Cnt
mGluR5
170KDa
130KDa
Actin
55KDa
40KDa

Figure 5a

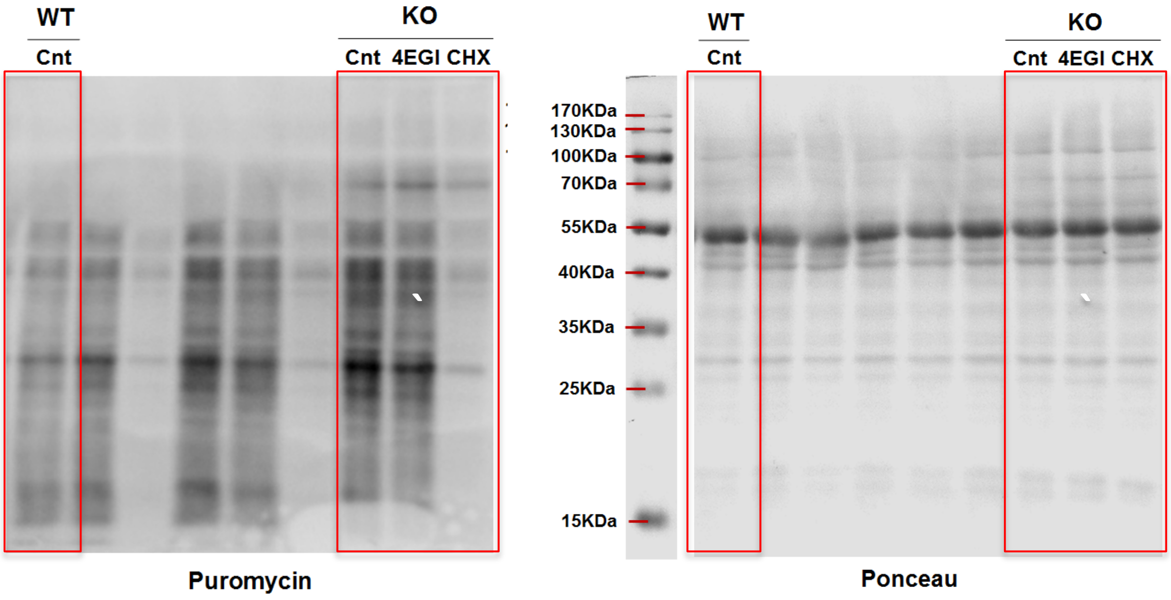

Figure 5b

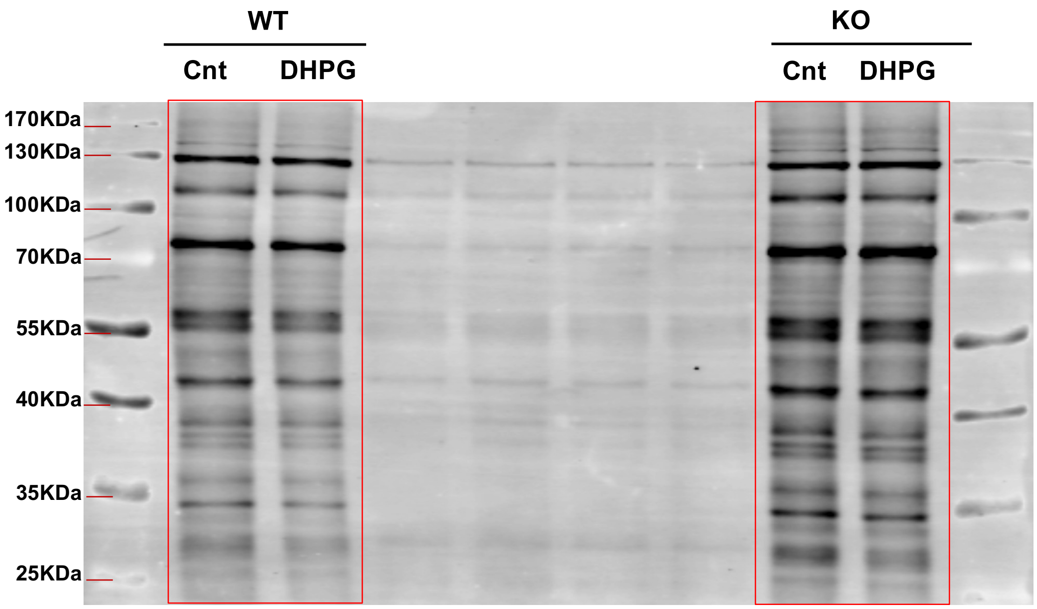

Figure 5c

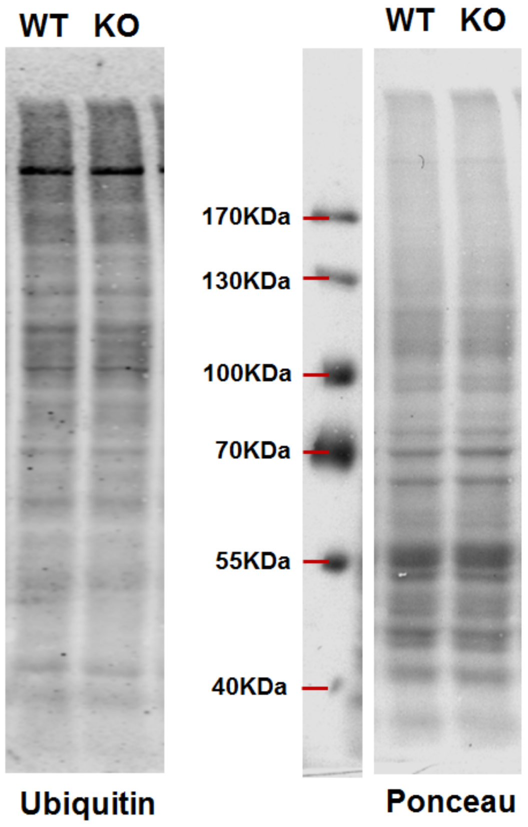
WT KO
WT KO
170KDa
130KDa
100KDa
70KDa
55KDa
40KDa
Ubiquitin
Ponceau

Figure 5d

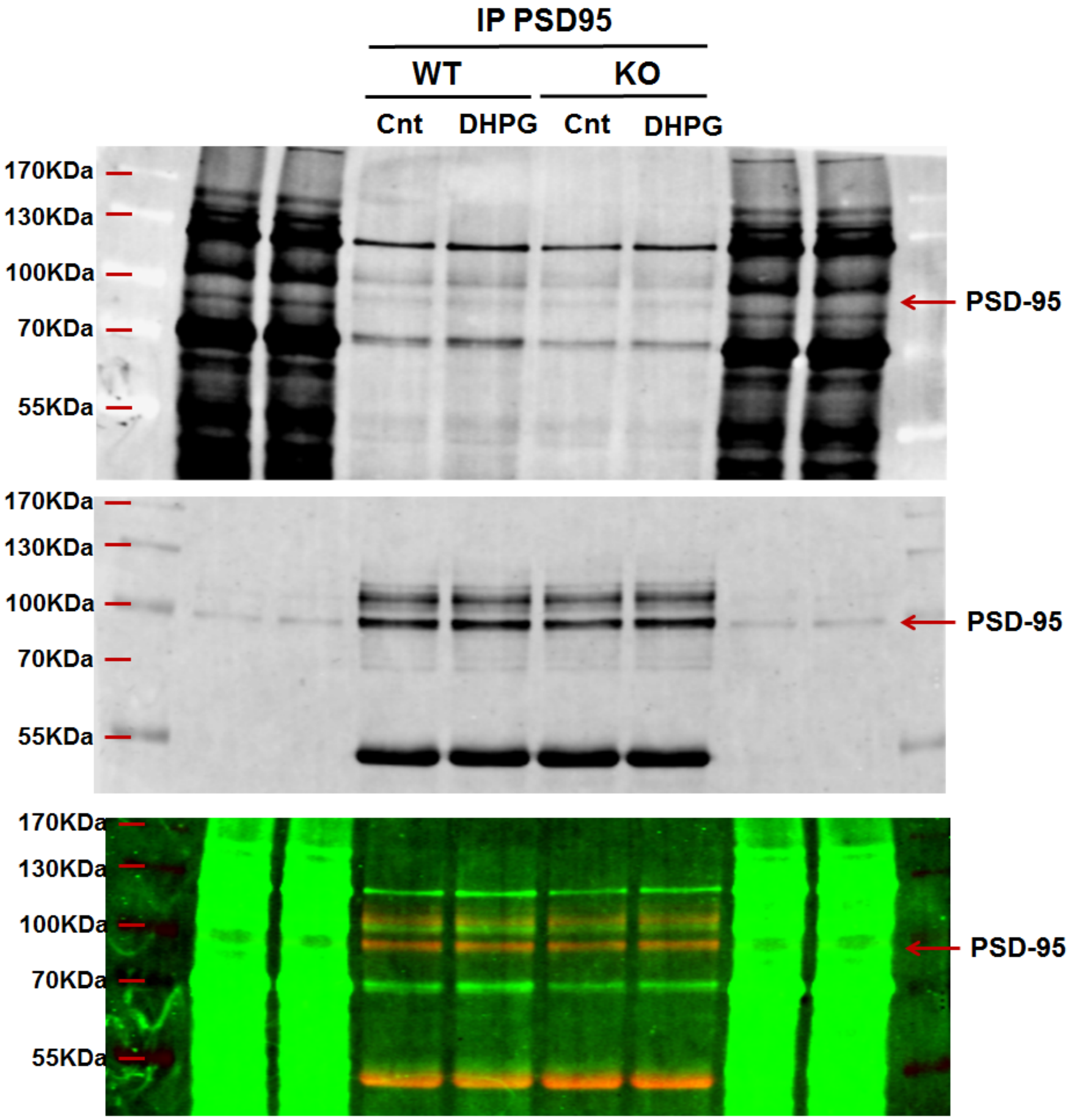

IP PSD95
WT
KO
Cnt
DHPG
Cnt
DHPG
170KDa
130KDa
100KDa
70KDa
55KDa
PSD-95
170KDa
130KDa
100KDa
70KDa
55KDa
PSD-95
170KDa
130KDa
100KDa
70KDa
55KDa
PSD-95

Figure 6c

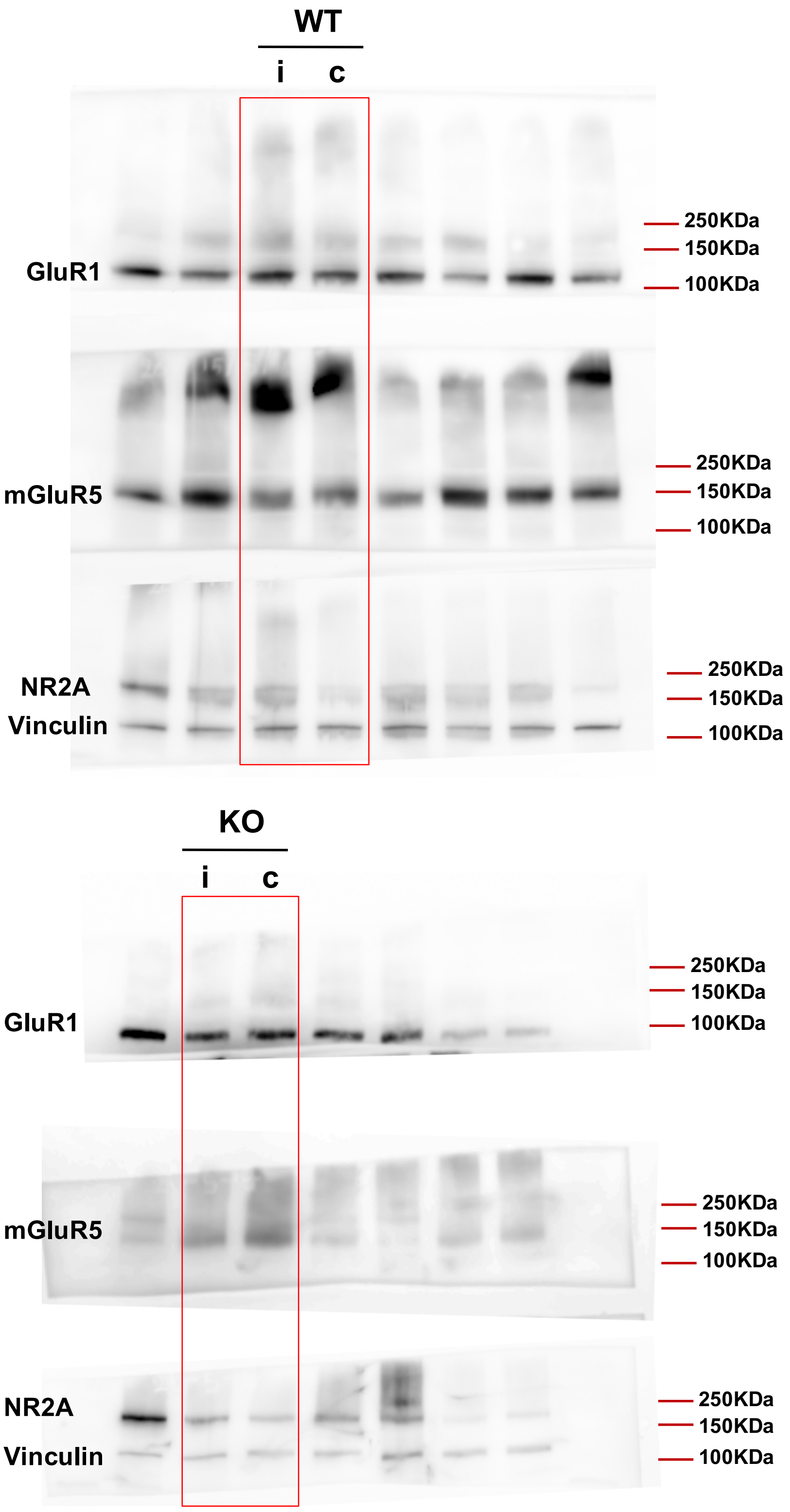
WT
i c
GluR1
mGluR5
NR2A
Vinculin
250KDa
150KDa
100KDa
KO
i c
GluR1
mGluR5
NR2A
Vinculin
250KDa
150KDa
100KDa

Figure S3a

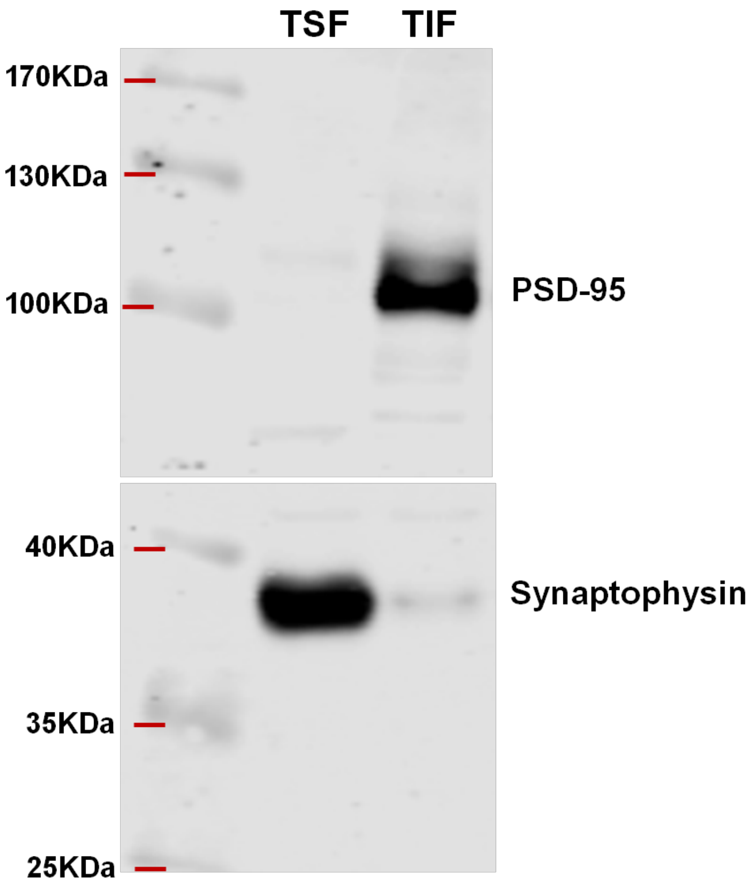
TSF
TIF
170KDa
130KDa
100KDa
PSD-95
40KDa
35KDa
25KDa
Synaptophysin

Figure S3b

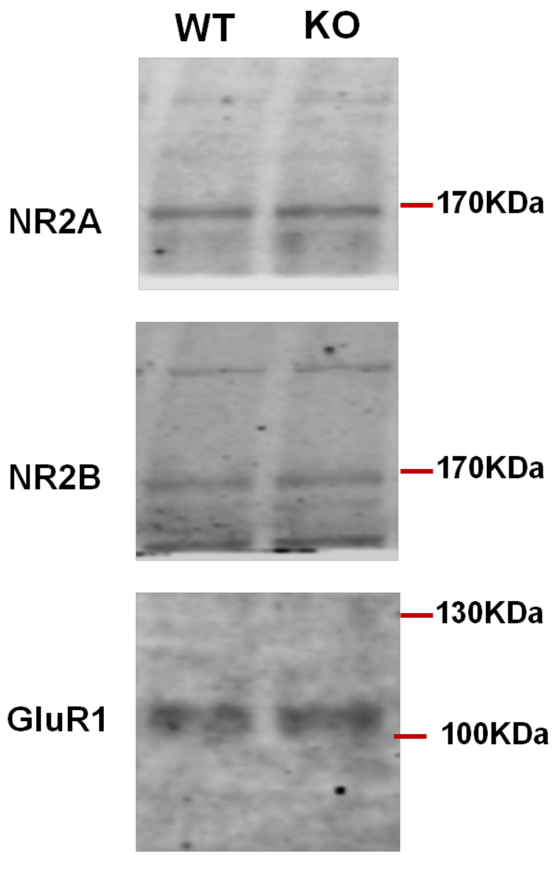
WT
KO
NR2A
170KDa
NR2B
170KDa
GluR1
130KDa
100KDa
GluR2
130KDa
100KDa
mGluR5
170KDa
130KDa
Actin
55KDa
40KDa

Figure S3c

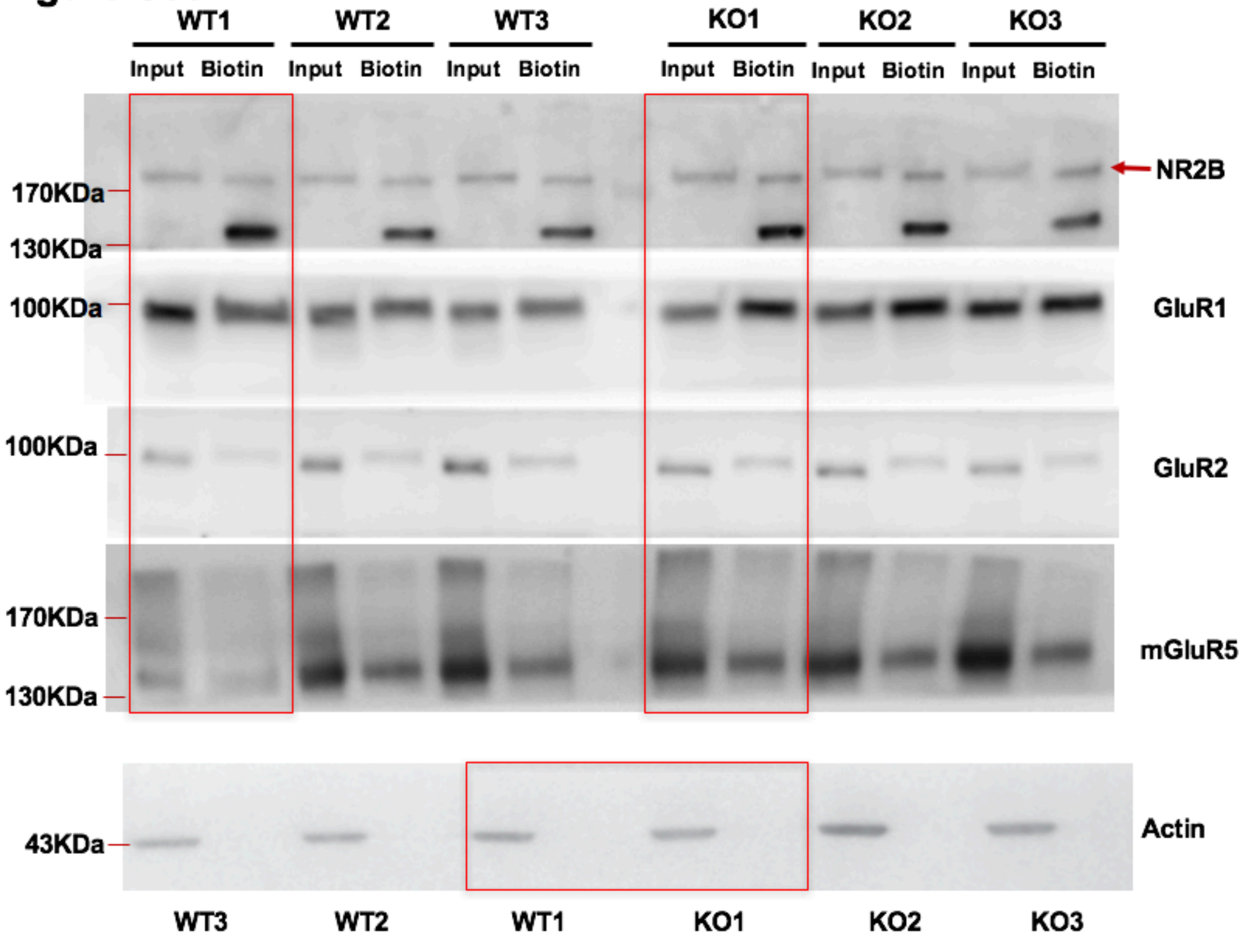

Figure S3d

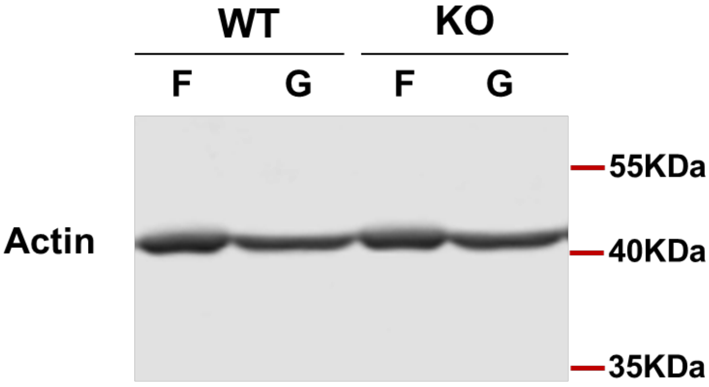

Supplementary Table 1. Behavioral phenotypes of *BC1* KO and two mouse models of IDs (*Fmr1* KO and *Cyfip1* +/-).

Behavioral task	*BC1* KO mouse	*Fmr1* KO mouse	*CYFIP1* Het mouse
Open field	Increased or no anxiety[2,a]<br>Normal activity[2,a]	Increased or decreased anxiety[3,4]<br>Hyperactivity[3]	Normal[5]
Elevated plus/zero maze	Increased anxiety[2]	Increased or decreased anxiety[3]	Normal[5]
Marble burying	Normal[a]	Normal[6]	-
Passive avoidance	-	Normal or decreased[3]	Enhanced Extinction[5]
Active avoidance	Mild decreased[7]	Mild decreased[7]	-
Fear conditioning	-	Normal or decreased[3]	Normal[5]
Morris water maze	Normal[2]	Normal[3]	Normal[5]
Barnes maze	Decreased number of errors[2]	Decreased memory[3]	-
Radial maze	Normal[2]	Normal[3]	-
Y/T-maze	Normal[2]	Decreased alternation[3]	-
Novel object recognition (NOR)	Decreased[a]	Normal or decreased[3,8]	-
Texture NOR	Decreased[a]	Decreased[8]	-
Sociability	Decreased[a]	Normal[3]	Normal[5]
Social novelty	Normal[a]	Decreased[3]	-
Tube test	Normal[a]	Increased dominance[9]	-
Audiogenic seizures	Increased[7,10]	Increased[3,7]	-
Self-grooming	Normal[a]	Increased[11,12]	-
Nest building	Normal[a]	Decreased[13]	-

[a]This work. The table provides a representative but not exhaustive overview of the published literature.

REFERENCES

1. Zalfa, F., *et al.* The fragile X syndrome protein FMRP associates with BC1 RNA and regulates the translation of specific mRNAs at synapses. *Cell* **112**, 317-327 (2003).
2. Lewejohann, L., *et al.* Role of a neuronal small non-messenger RNA: behavioural alterations in BC1 RNA-deleted mice. *Behav Brain Res* **154**, 273-289 (2004).
3. Santos, A.R., Kanellopoulos, A.K. & Bagni, C. Learning and behavioral deficits associated with the absence of the fragile X mental retardation protein: what a fly and mouse model can teach us. *Learn Mem* **21**, 543-555 (2014).
4. Restivo, L., *et al.* Enriched environment promotes behavioral and morphological recovery in a mouse model for the fragile X syndrome. *Proc Natl Acad Sci U S A* **102**, 11557-11562 (2005).
5. Bozdagi, O., *et al.* Haploinsufficiency of Cyfip1 produces fragile X-like phenotypes in mice. *PLoS One* **7**, e42422 (2012).
6. Spencer, C.M., *et al.* Modifying behavioral phenotypes in Fmr1KO mice: genetic background differences reveal autistic-like responses. *Autism Res* **4**, 40-56 (2011).
7. Zhong, J., *et al.* Regulatory BC1 RNA and the fragile X mental retardation protein: convergent functionality in brain. *PLoS One* **5**, e15509 (2010).
8. Orefice, L.L., *et al.* Peripheral Mechanosensory Neuron Dysfunction Underlies Tactile and Behavioral Deficits in Mouse Models of ASDs. *Cell* **166**, 299-313 (2016).
9. de Esch, C.E., *et al.* Fragile X mice have robust mGluR5-dependent alterations of social behaviour in the Automated Tube Test. *Neurobiol Dis* **75**, 31-39 (2015).
10. Zhong, J., *et al.* BC1 regulation of metabotropic glutamate receptor-mediated neuronal excitability. *J Neurosci* **29**, 9977-9986 (2009).
11. Mines, M.A., Yuskaitis, C.J., King, M.K., Beurel, E. & Jope, R.S. GSK3 influences social preference and anxiety-related behaviors during social interaction in a mouse model of fragile X syndrome and autism. *PLoS One* **5**, e9706 (2010).
12. McNaughton, C.H., *et al.* Evidence for social anxiety and impaired social cognition in a mouse model of fragile X syndrome. *Behav Neurosci* **122**, 293-300 (2008).
13. Pasciuto, E., *et al.* Dysregulated ADAM10-Mediated Processing of APP during a Critical Time Window Leads to Synaptic Deficits in Fragile X Syndrome. *Neuron* **87**, 382-398 (2015).
